# Acute-phase Serum Cytokine Levels and Correlation with Clinical Outcomes in Children and Adults with Primary and Secondary Dengue Virus Infection in Myanmar between 2017 and 2019

**DOI:** 10.3390/pathogens11050558

**Published:** 2022-05-09

**Authors:** Khine Mya Nwe, Mya Myat Ngwe Tun, Theingi Win Myat, Chris Fook Sheng Ng, Moh Moh Htun, Htin Lin, Nang Sarm Hom, Aung Min Soe, Annie Elong Ngono, Shinjiro Hamano, Kouichi Morita, Kyaw Zin Thant, Sujan Shresta, Hlaing Myat Thu, Meng Ling Moi

**Affiliations:** 1 Department of Virology, Institute of Tropical Medicine, Nagasaki University, Nagasaki 852-8523, Japan; drkhinemyanwe@gmail.com (K.M.N.); myamyat@tm.nagasaki-u.ac.jp (M.M.N.T.); dr.aungminnsoe@gmail.com (A.M.S.); moritak@nagasaki-u.ac.jp (K.M.); 2 Program for Nurturing Global Leaders in Tropical and Emerging Communicable Diseases, Graduate School of Biomedical Sciences, Nagasaki University, Nagasaki 852-8501, Japan; chrisng@m.u-tokyo.ac.jp (C.F.S.N.); shinjiro@nagasaki-u.ac.jp (S.H.); 3 Department of Medical Research, Yangon 11191, Myanmar; drtheingiwinmyat@gmail.com (T.W.M.); mohmoh.htun@gmail.com (M.M.H.); drhtinlin@gmail.com (H.L.); sarmhom@gmail.com (N.S.H.); drkz.thant@gmail.com (K.Z.T.); 4 Department of Pathology, North Okkalapa General and Teaching Hospital, Yangon 11031, Myanmar; 5 School of International Health, Graduate School of Medicine, University of Tokyo, Tokyo 113-0033, Japan; 6 Center for Infectious Disease and Vaccine Research, La Jolla Institute for Immunology, La Jolla, CA 92037, USA; aelong@lji.org; 7 Department of Parasitology, Institute of Tropical Medicine, Nagasaki University, Nagasaki 852-8523, Japan

**Keywords:** dengue, Myanmar, cytokines, clinical severity

## Abstract

The dengue virus (DENV) has been endemic in Myanmar since 1970, causing outbreaks every 2–3 years. DENV infection symptoms range from mild fever to lethal hemorrhage. Clinical biomarkers must be identified to facilitate patient risk stratification in the early stages of infection. We analyzed 45 cytokines and other factors in serum samples from the acute phase of DENV infection (within 3–5 days of symptom onset) from 167 patients in Yangon, Myanmar, between 2017 and 2019. All of the patients tested positive for serum DENV nonstructural protein 1 antigen (NS1 Ag); 78.4% and 62.9% were positive for immunoglobulin M (IgM) and G (IgG), respectively; and 18.0%, 19.8%, and 11.9% tested positive for serotypes 1, 3, and 4, respectively. Although the DENV-4 viral load was significantly higher than those of DENV-1 or DENV-3, disease severity was not associated with viral load or serotype. Significant correlations were identified between disease severity and CCL5, SCF, PDGF-BB, IL-10, and TNF-α levels; between NS1 Ag and SCF, CCL5, IFN-α, IL-1α, and IL-22 levels; between thrombocytopenia and IL-2, TNF-α, VEGF-D, and IL-6 levels; and between primary or secondary infection and IL-2, IL-6, IL-31, IL-12p70, and MIP-1β levels. These circulating factors may represent leading signatures in acute DENV infections, reflecting the clinical outcomes in the dengue endemic region, Myanmar.

## 1. Introduction

The dengue virus (DENV) is a mosquito-borne arbovirus that is endemic in more than 130 countries and represents a significant public health burden worldwide [1]. The first DENV outbreak in Myanmar was reported in 1970; moreover, a particularly sharp increase in infections has been noted in the past 10 years despite concentrated vector-control efforts. Myanmar is currently classified by the World Health Organization (WHO) as a high-burden country for DENV in the Asia-Pacific region [2,3].

DENV is a member of the *Flaviviridae* family of single-stranded RNA viruses and exists as 4 antigenically distinct serotypes: DENV-1–4. Infection with one serotype does not protect against infection against a heterotypic serotype, and secondary infections carry an elevated risk of developing severe dengue. Depending on the severity of clinical features, symptomatic DENV infection is categorized by WHO as dengue without warning signs (DWoWS), dengue with warning signs (DWWS), and severe dengue (SD) [4]. Factors that determine the severity of illness following DENV infection are still elusive. The known contributors are viral serotype and genotype, and various aspects of the host immune response. Regarding the immune response, patients with secondary DENV infection are at an increased risk of the development of SD owing to a phenomenon known as antibody-dependent enhancement of infection. Additionally, DENV infection can induce increases in the production of cytokines and other inflammatory mediators. Cytokines, as proteinaceous molecules, are secreted during innate and adaptive immune responses that disrupt physiological functions such as hemostatic regulation, which leads to many features associated with SD, including plasma leakage, shock, and hemorrhage. Indeed, levels of several mediators have been used as stratification and prognostic markers for patients with DENV [5]. The development of severe disease or full recovery related with the participation of cytokines in the pathogenesis. [6]. Dengue is known to infect various immune cells, including dendritic cells and monocyte and hence produce inflammatory and antiviral cytokines leading to tissue and organ damage. Meanwhile, these proteins express inflammatory responses and modulate several hemorrhagic and hemostatic regulations and may act as markers for patient stratification and prognosis [5,7].

In this study, we investigated the levels of 45 cytokines, chemokines, and growth factors in acute-phase serum samples collected from a cohort of DENV-infected individuals in Myanmar. We then determined whether significant correlations existed between acute-phase cytokine levels and disease severity as well as other relating clinicopathological factors on both viral and host aspects to support our findings.

## 2. Materials and Methods

### 2.1. Study Population and Samples

This was a cross-sectional study of children and adults from two hospitals in Yangon, a DENV-endemic region of Myanmar, between June 2017 and December 2019. The sample size was calculated using the following formula:

(1) $$n=\frac{Z_{1-\frac{\partial }{2}}^{2}\sigma^{2}}{d^{2}}$$

minimal required sample size was 156 ± 8 assuming 95% confidence interval. A total of 167 serum samples were collected during the acute phase (between 3–5 days after onset of fever). Laboratory tests included hematocrit, platelet count, and an initial rapid nonstructural protein 1 antigen (NS1 Ag) test (SD Bioline Dengue NS1 Ag; Standard diagnostic Inc., Suwon, Korea). Patients were classified according to the 2009 WHO guideline as having DWoWS, DWWS, and SD based on clinical findings and laboratory tests. For cytokine analysis, the control serum samples were collected from 10 healthy individuals who were confirmed to be negative for NS1 Ag, anti-DENV immunoglobulin (Ig)M, anti-DENV IgG, and anti-Zika virus IgG. There were no records of underlying diseases in any of the patients in this study.

### 2.2. Ethical Approval

This study was approved by the Ethics Review Committee on Medical Research Including Human Subjects at the Department of Medical Research, Myanmar (Ethics/DMR/2017/068) and the Institute of Tropical Medicine at Nagasaki University in Nagasaki, Japan (170707205).

### 2.3. Serology and NS1 Ag Detection

All of the serum samples, qualitatively screened for NS1 Ag using the rapid diagnostic test were analyzed using qualitative/semi-quantitative PLATELIA^TM^ DENV NS1 Ag enzyme-linked immunosorbent assay (ELISA) kits (Bio-Rad, Hercules, CA, USA) according to the manufacturer’s instructions. NS1 Ag levels are expressed as the ratio of the OD at 450/620 nm of the sample to that of the kit calibrator cutoff value. Ratios of <0.5, 0.5–1.00, and >1.00 were considered negative, equivocal, and positive, respectively. Samples were tested for the presence of anti-DENV antibodies using previously described dengue IgM capture ELISA [8] and dengue IgG indirect ELISA and optical density (OD) was measured at 497 nm. Serum samples were considered positive for IgM antibody when P/N ratio was ≥2 and IgG antibody titer was >3000. If the IgG titer was ≥29,000, infection was considered as secondary dengue, whereas a titer of <29,000, was considered as primary dengue [9,10,11].

### 2.4. Quantification of DENV Genomic RNA and Serotyping

RNA was extracted from 140 μL of serum using a viral RNA mini-kit (Qiagen, Hilden, Germany), according to the manufacturer’s protocol. For quantitative reverse-transcription polymerase chain reaction (qRT-PCR), 5 μL of RNA was used and the envelope gene was amplified in a total of 20 μL of the reaction mixture (5 μL TaqMan master mix, 9 μL nuclease-free water, 0.3 μL of 100 pmol forward and reverse primers, 0.4 μL of DENV serotype-specific primer) using TaqManFast Virus 1-Step MasterMix (Life Technologies, Carlsbad, CA, USA). Serotype specific primers and probes are described in (Supplementary Table S1). Ten-fold serial dilutions of standard RNA (10^2^–10^8^ genome copies) were amplified for quantification of viral genome copies [12,13]. The detection limit was 100 genome copies, and the results are expressed as log10 genome copies/mL of serum [13].

### 2.5. Identification and Quantification of Cytokines

A total of 45 cytokines, chemokines, and growth factors were measured using a Cytokine/Chemokine/Growth Factor 45-Plex Human ProcartaPlex Panel 1 kit (Invitrogen, Carlsbad, CA, USA) according to the manufacturer’s protocol. Fluorescence signals were detected using the Bio-plex 200 system (Bio-Rad, Hercules, CA, USA), and the data were analyzed using the Bio-plex Manager 6.2 software (Bio-Rad, Hercules, CA, USA). Results that were outside the lowest range of the standard curve were set as half the lowest value in the set for the respective analyte. The value that was outside the highest range was replaced with the highest value in the set [14].

### 2.6. Statistical Analysis

Data were analyzed using SPSS for Windows version 16.0 (IBM Corp., Armonk, NY, USA) and GraphPad Prism version 9.0.0 (GraphPad Software, San Diego, CA, USA). Continuous variables are presented as the median and interquartile range (IQR) or mean ± standard deviation. Skewness was tested to assess the normal distribution of the variables. As the data did not show normal distribution, we used non-parametric tests. Data from more than two groups were compared using MANCOVA, and between two groups were compared using the Mann–Whitney U test based on distribution of data. The Spearman’s test was used to identify correlations and the results are presented as the coefficients (ρ −1 to +1). *p*–values of < 0.05 were considered as statistically significant. PCA was conducted in R (Version 4.1.2) with plotting tools from the *factoextra* package.

## 3. Results

### 3.1. Clinical Presentation of the Study Population

A total of 167 children and adults with confirmed DENV infection (serum NS1 Ag-positive) were enrolled in the study. Serum samples were collected from all of the participants between 3–5 days after the onset of fever. The majority of patients were male (*n* = 106, 63.5%) and aged between 6–12 years (*n* = 101, 60.5%). Based on the WHO dengue criteria, 64 (38.3%), 64 (38.3%), and 39 (23.3%) patients were classified as having DWoWS, DWWS, and SD, respectively (Table 1).

**Figure 1:**
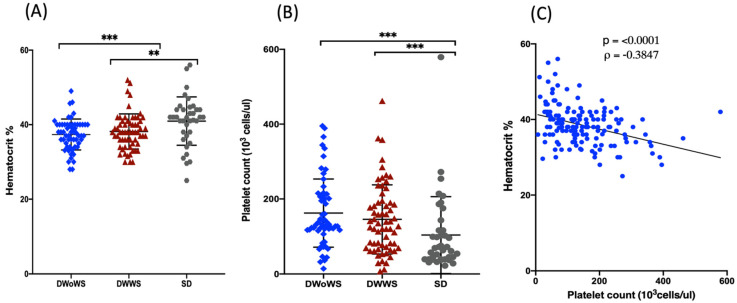
Hematocrit and platelet count in dengue patients stratified according to disease severity. (**A**) Hematocrit (%) and (**B**) platelet count (10^3^ cells/μL) in patients with DWWS (*n* = 64, 38.3%), DWoWS (*n* = 64, 38.3%), and SD (*n* = 39, 23.3%). (**C**) Spearman’s rank correlation (⍴) between hematocrit and platelet counts. ** *p* < 0.01, *** *p* < 0.001 determined using the Mann–Whitney U test.

All of the study participants presented with fever and were positive for capillary fragility, as determined using the tourniquet test. Vomiting (*n* = 70, 41%) and abdominal pain (*n* = 45, 26%) were the most common symptoms, followed by gastrointestinal and bleeding manifestations. Seizures were only observed three patients (Supplementary Figure S1). Hematocrit values were significantly higher in the SD group (40.9%) than in either the DWWS group (38.2%, *p* < 0.001) or the DWoWS group (37.4%, *p* < 0.001; Figure 1A). Conversely, the platelet count was significantly lower in the SD group compared with that in both the DWoWS and DWWS groups (Figure 1B). There was a significant negative correlation between the platelet count and hematocrit (Figure 1C).

### 3.2. Serological Analyses

All 167 patients were positive for NS1 Ag. A total of 132 patients (78.5%) were positive for anti-DENV IgM, of whom 50, 48, and 33 were in the DWoWS, DWWS, and SD groups, respectively. Similarly, 105 (62.9%) patients were positive for anti-DENV IgG, of whom 41, 31, and 33 were in the DWoWS, DWWS, and SD groups, respectively (Table 1). Of the 83 patients positive for DENV RNA as determined using qRT-PCR, 32 (50%), 33 (51.5%), and 18 (46.1%) patients in DWoWS, DWWS and SD groups, respectively (Table 1).

**Figure 2:**
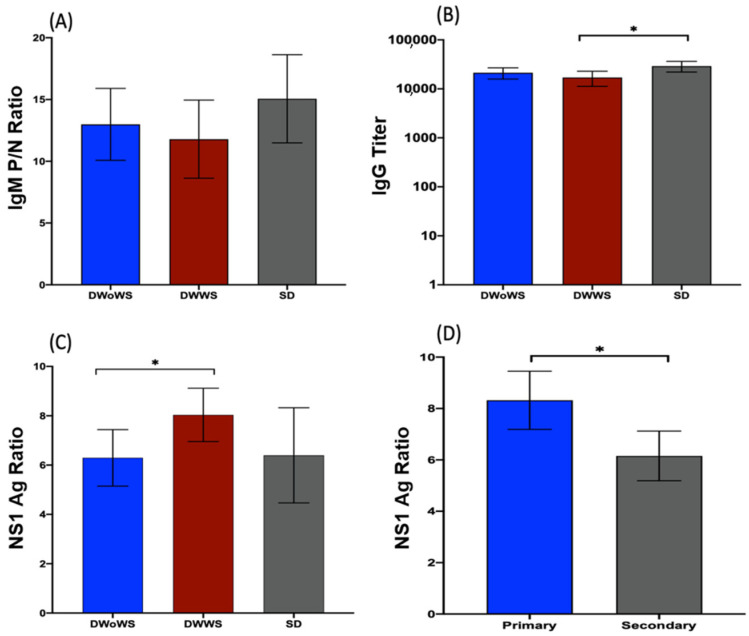
Serological analysis of dengue patients during the acute phase of infection. (**A**–**C**) IgM P/N ratio (**A**), IgG titer (**B**), and mean NS1 Ag ratio (**C**) in patients in the DWoWS, DWWS, and SD groups. (**D**) Mean NS1 Ag ratio in patients with primary infection (*n* = 64, 38.3%) and secondary infection (*n* = 103, 61.6%). * *p* < 0.05 determined using the Mann–Whitney U test.

We detected no significant differences between the DWoWS, DWWS, and SD groups for the IgM P/N ratio (Figure 2A) or NS1 Ag ratio (Figure 2C). However, the mean IgG titer was significantly higher in the SD group than in the DWWS group, but not than that in the DWoWS group (Figure 2B), and the NS1 Ag ratio (level) was significantly higher in patients with serologically determined primary infection vs. secondary infection (Figure 2D).

### 3.3. Virus Serotypes and Viremia

**Figure 3:**
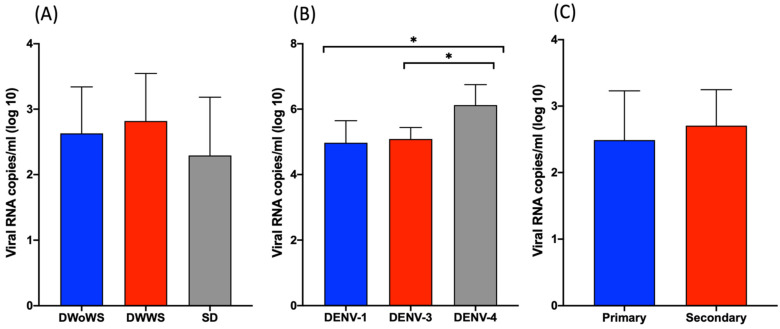
Viremia in dengue patients during the acute phase of infection. (**A**–**C**) Mean viral RNA load (copies/mL) in serum samples according to (**A**) disease severity, (**B**) DENV serotype, and (**C**), primary or secondary infection status. * *p* < 0.05 determined using the Mann–Whitney U test.

Among the 83 patients who were positive for viral RNA as determined using qRT-PCR analysis, 30 (36.1%) were infected with DENV-1, 33 (39.7%9) with DENV-3, and 20 (24.1%) with DENV-4. There were no significant differences in DENV viral load between patients with different disease severity (Figure 3A) or those with primary vs. secondary infection (Figure 3C). However, patients infected with DENV-4 had significantly higher levels of viremia than those infected with DENV-1 or DENV-3 (Figure 3B).

### 3.4. Serum Levels of Inflammatory Cytokines, Chemokines, and Growth Factors

**Figure 4:**
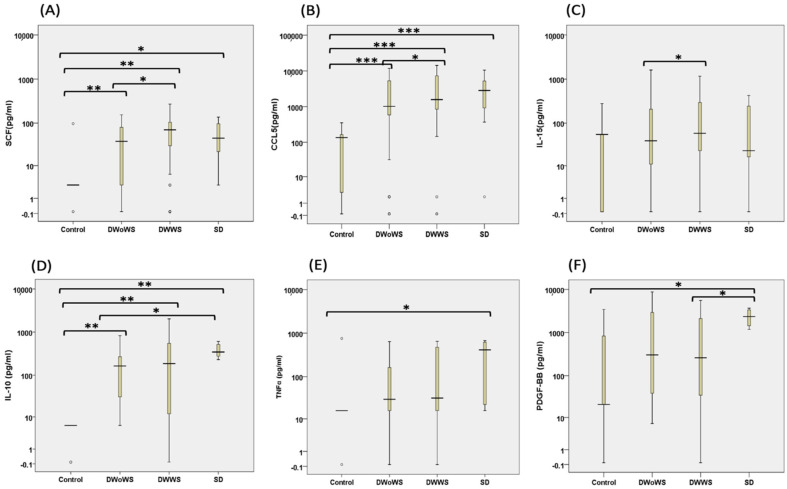
Serum cytokine levels in dengue patients during the acute phase of infection. (**A**–**F**) Cytokine levels in serum samples from healthy individuals (Control; *n* = 10) or patients with DWoWS (*n* = 64), DWWS (*n* = 64), and SD (*n* = 39). (**A**) SCF, (**B**) CCL5, (**C**) IL-15, (**D**) IL-10, (**E**) TNF-⍺, and (**F**) PDGF-BB. Circles represent samples outliers to the median value. Box plots show the median and interquartile range. * *p* < 0.05, ** *p* < 0.01, *** *p* < 0.001 using MANCOVA (after controlling for Age, gender, days of fever and IgM) the Mann–Whitney U test.

Levels of 45 cytokines, chemokines, and growth factors in serum samples obtained from the 167 confirmed dengue patients collected between day 3–5 of fever onset, and 10 healthy individuals were analyzed using multiplexed immunoassay. Of the factors evaluated, several were present at significantly different levels between the patient and healthy control groups as well as between the patient severity subgroups while age, gender, days of fever and IgM had no effect on serum cytokines level. For example, levels of SCF, CCL5, and interleukin (IL)-15 were significantly higher in the DWWS group than in the DWoWS group, and chemokine ligand 5 (CCL5) and stem cell factor (SCF) levels were significantly higher in the DWWS, DWoWS, and SD groups than in the control group (Figure 4A–C). Of note, levels of IL-10, tumor necrosis factor-α (TNF-α), and platelet-derived growth factor (PDGF-BB) were particularly high in samples from patients in the SD group (Figure 4D–F).

**Figure 5:**
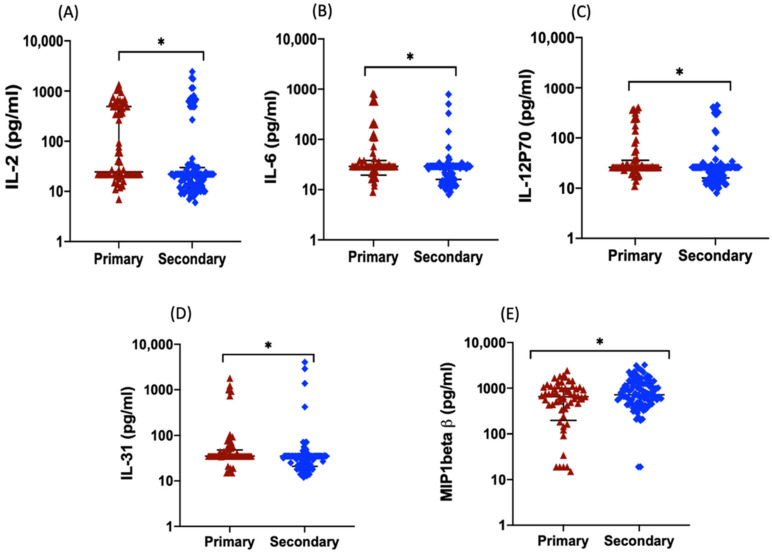
Serum cytokine levels in dengue patients stratified by primary and secondary infection. (**A**) IL-2, (**B**) IL-6, (**C**) IL-31, (**D**) IL-12p70, and (**E**) MIP-1β. * *p* < 0.05 as determined by using the Mann–Whitney U test.

In addition, levels of 16 mediators IL-1α, IL-1β, IL-5, IL-7, IL-8 (CXCL8), IL-13, IL-17A, PIGF1 (placental growth factor), IL-23, IP10 (CXCL10), MIP-1β, NGF-β, EGF, vascular endothelial growth factor D (VEGF-D), FGF-2 (fibroblast growth factor) and CCL2, were significantly higher in patients compared with those in healthy subjects, but the levels were not significantly different between the SD, DWoWS, and DWWS groups (Table 2). Levels of MIP-1α, SDF-1α, IL-27, LIF, IL-2, IL-6, IL-18, IL-21, IL-22, CCL11, IL-12p70, IL-1RA, interferon (IFN)-γ, HGF, and GM-CSF were not significantly different among the three dengue disease groups or between the healthy subjects and dengue patient groups. MIP-1β levels were significantly higher in patients with secondary infection compared with those with primary infection, whereas the reverse was true for IL-2, IL-6, IL-31, and IL-12p70 levels (Figure 5). IL-2, IL-6, TNF-α, and VEGF-D levels correlated negatively with platelet counts. IFN-α, IL-1α, SCF, and CCL5 levels positively correlated with NS1 Ag levels, whereas IL-22 levels correlated negatively with NS1 Ag levels (Table 3). Principal Component Analysis (PCA) was conducted to explore the potential clustering of cytokine levels among the 167 patients.

**Figure 6:**
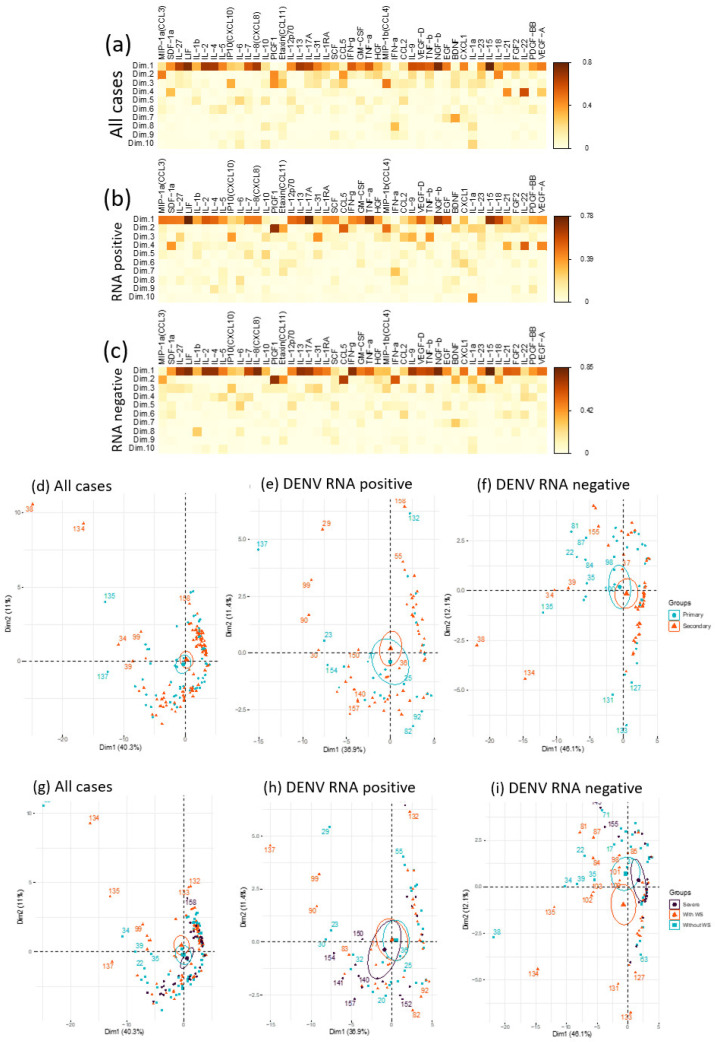
Principal component analysis (PCA) of different cytokine levels from dengue patients with severe and non-severe dengue. The contributions of cytokines to the top principal components (Dim.1 to Dim10) in all cases (**a**), dengue cases that demonstrated virus RNA (**b**) and in the absence of virus RNA (**c**). Clustering of individuals by primary and secondary infection according to principal component 1 (Dim.1) and component 2 (Dim.2) (**d**–**f**). Clustering of individuals by the severity of infection—severe, with and without warning sign (WS) according to principal component 1 (Dim.1) and 2 (Dim.2) for all cases (**g**), cases with positive RT-PCR (*n* = 83, (**h**)), and cases with negative RT-PCR (*n* = 84, (**i**)). Circles represent the group means. All cases (*n* = 167) were used in the analyses (**a**–**i**).

A total of 3 principal components were extracted, accounting for 60.8% of variation in the data (Figure S3). In terms of percentage contribution, there was no clear dominance by any cytokine in the first component despite the largest explained variance at 40.3% (Figure S4). The second and third component exhibited some overlap with considerable contributions from similar cytokines such as PIGF1 and CCL5 (Figure 6a–c and Figure S4). Based on this, the comparisons between components 2 and 3 were omitted in subsequent analyses. Additional subgroup analyses separating cases with positive and negative for DENV RNA were also performed (Figure 6d–i). The extracted principal components were first analyzed for the capacity of distinguish between primary and secondary infection cases and by disease severity (Figure 6d–f and Figure S5). While there was limited PCA distinction between the primary and secondary infections based on the selected components (Figure 6d–f), the data suggest that the first two principal components were able to distinguish cases by disease severity (Figure 6g–i and Figure S6), particularly among those that were negative for DENV RNA within different group means by severity (Figure 6i).

## 4. Discussion

In this study, we characterized the acute-phase serological status of 167 children and adults with dengue in Yangon, Myanmar, between 2017–2019, and correlated the findings with disease severity. Nearly 40% of the patients were characterized as having DWWS and DWoWS, whereas only about 20% had SD. The proportion of SD patients in our study is smaller than that detected in another study of DENV-infected patients in Yangon during the same period [15]. The most common presenting symptoms in the patients in our study were fever, vomiting, and headache, and both the frequency and type of symptoms were similar to those described in other studies of DENV infection in India [16,17].

We observed several noteworthy differences in acute-phase parameters between patients with DWWS, DWoWS, and SD. Hematocrit values were significantly higher and platelet counts were significantly lower in the patients in the SD group compared with those in the DWoWS and DWWS groups. Platelet count presentations during DENV were similar to those observed in a population of two widely separated regions in Myanmar in 2015 [18,19]. Platelets help to maintain hemostasis, facilitate thrombosis, angiogenesis [19]. According to WHO guidelines, the clinical parameters of hematocrit and platelet counts are useful for categorization, which was confirmed in this study.

The majority of our patient group (103 (61.6%)) had secondary DENV infections, which is a similar proportion to the previous study of DENV infection in this region, Yangon, Myanmar between 2017–2019 [15]. While nationwide monitoring has been performed in Myanmar, there were limited information on the characteristics of serotypes and genotypes and associated clinical outcomes. In Myanmar, hospital-based diagnosis was conducted by using either NS1 or IgM antibody rapid test kit, and hence national surveillance data, in the absence of DENV sequence and strain information, may not fully reflect the DENV situation in the region. With limited data on the DENV situation in Myanmar, our data reports the findings of clinical outcomes and cytokine patterns, in association with the emergence of newer DENV strains, DENV-3 genotype-I in Myanmar [15]. Although all of the patients showed positive to dengue screening test, NS1 Ag detection by RDT, 83 were documented as dengue RNA positive by real time RT-PCR. NS1 is a highly conserved glycoprotein that is present at high concentration in the sera of DENV-infected patients and related with severe dengue by causing endothelial damage. [20,21]. Some studies have indicated that RT-PCR detection of DENV nucleic acid declined with time indicating the suitability of RT-PCR during the viremic febrile phase [20,21]. The detection of NS1 is useful in the early stages of the disease, particularly during the period of days 3–5 after onset of the disease, when viremia levels may be below detection levels and anti-IgM [22].

DENV-1, -3, and -4 serotypes were detected in our patient cohort, and the viral load was significantly higher in patients infected with DENV-4 than with DENV-1 or -3; however, we observed serotype difference or viremia level have no effect on severe dengue. All DENV serotypes are known to cause severe clinical features, which have previously been reported to be dependent of the viral serotype and genotype [23]. The NS1 Ag level saw no significant changes in severe dengue in this study. However, another study mentioned that DENV NS1 antigen levels correlate with disease severity early in the dengue illness [20]. Aligning with other studies, we found that mean serum NS1 Ag levels were significantly higher in patients with primary infection as compared to those with secondary infection [6,24]. The lower NS1 Ag levels during secondary infection could result from the immune complex formation with circulating anti-DENV antibodies, which would prevent the detection of free NS1 [25].

The serum cytokine profiles of DENV-infected patients may provide valuable information for characterizing immunological response patterns associated with disease severity, and thus help to identify patient groups at risk for poorer outcomes. DENV infects various immune cells, causing alterations in inflammatory and antiviral cytokine levels, leading to tissue or organ damage [5]. Maintaining the balance between inflammation and anti-inflammation is critical for infection control. The inflammatory response and production of cytokines play key roles in the development of severe clinical manifestations in response to viral infection, where a rapid increase in cytokine levels seems to be the predominant factor driving disease severity [26]. In the present study, levels of TNF-α, IL-10, and PDGF-BB were higher in patients with SD compared with those in patients with DWWS or DWoWS. A study has linked high levels of TNF-α, a prototypical pro-inflammatory, and antiviral cytokine, to increased risk of SD [27]. An in-vitro experiment, synergistic effect of TNF-α and DENV infection induced permeability changes in endothelial cells [28]. One of the in-vivo experiment also reported that TNF-α contributed lethal effect on AG129 mice [29]. However, this association was not observed in other studies [30,31]. Despite the beneficial ability of TNF-α to inhibit DENV replication, high levels of this cytokine may have detrimental effects on endothelial cells, resulting in increased vascular permeability. In our study, we observed that TNF-α levels were inversely correlated with platelet counts, which may suggest another link to the hemorrhagic features of dengue. This finding is consistent with other studies conducted in India and Pakistan [32,33,34]. Matching with previous reports, patients with severe dengue exhibited an increased level of IL-10 compared to the levels in patients with mild disease [35,36]. IL-10 is a key immunoregulatory cytokine and is produced by many cell types, including monocytes/macrophages, dendritic cells, and regulatory T cells. Genetic polymorphisms in the IL-10 gene have been associated with the severity of the disease. IL-10 production in DENV-infected individuals may be exacerbated by antibody-dependent enhancement of infection [36]. However, IL-10 also plays a negative regulatory role in the immune response [37]. Consistent with our findings, PDGF-BB also significantly increased during febrile in another study [38].

We observed that levels of SCF, CCL5, and IL-15 were significantly higher in patients with DWWS than in those with DWoWS. SCF is expressed by fibroblasts and endothelial cells and plays important role in maintaining hematopoietic stem cells and hematopoiesis [39]. Levels of CCL5 were also higher in the DENV-infected patients relative to that in the healthy controls [40], which may be directly disease-relevant considering that CCL5 can enhance permeability and plasma leakage [41]. The higher IL-15 levels observed in dengue patients may contribute to an expansion of cytolytic natural killer cells, which can have both antiviral and tissue-damaging effects [30]. In the present study, we detected significantly higher levels of IL-1β, IL-8, IL-13, IP10, MIP-1β, and VEGF-D in the patient (DWoWS, DWWS, and SD) groups compared with those in the healthy control group, and the same patterns were seen in previous reports [17,27,34,41,42]. Among these, IL-8 and IL-1β have been demonstrated to play roles in plasma leakage [27]. DENV infected endothelial cells also secrete IP10, which is known to enhance vascular permeability and plasma leakage [33,43]. Endothelial cells can alter their permeability in response to those pro-inflammatory cytokines [42]. The elevated levels of MIP-1β seen in our patient cohort have also been noted previously, and this cytokine has been used as a marker of SD in a previous report [5]. Previous reports have described that VEGF levels are elevated in the sera of dengue infected mice compared to naïve mice [29].

The elevation in VEGF levels is likely linked to its role in angiogenesis and regulation of vascular permeability [44]. Comparing cytokine levels between patients with primary and secondary DENV infections, we observed that IL-2, IL-6, IL-31, and IL-12p70 were significantly higher in primary infection and MIP1β was significantly higher in secondary infection. It differed from the findings of another study that detected no significant difference between IL-6 levels in patients with primary vs secondary infections [45]. We identified significant correlations between serum IL-2, IL-6, TNF-α, and VEGF-D levels and thrombocytopenia in DENV-infected patients in the present study, consistent with the findings of other reports [6,32,42]. Thrombocytopenia, one of the most important factors in bleeding manifestation in patients with severe dengue, results from bone marrow suppression and increased destruction, such as platelet consumption during coagulopathy, complement activation, and greater peripheral sequestration [46]. Increased SCF, CCL5, IFN-α, and IL-1α levels and a decreased IL-22 level were associated with higher NS1 Ag levels in the present study. Although we did not detect an association between IL-10 and NS1 Ag levels, this has been suggested in another study of patients in Columbo, Sri Lanka [47]. Such differences in cytokine expression levels between studies could be the result of virus genetic composition, leading to differences in host response or, other differences in the host populations [48].

## 5. Conclusions

This study was first document of serum cytokine profiles during the acute phase of DENV infection in Myanmar pediatric and adult patients. Understanding changes in cytokine profiles early during DENV infection may be valuable for identifying patients at risk for the development of SD. Indeed, we found that levels of certain cytokines were significantly associated with disease severity; specifically, PDGF-BB, IL-10, and TNF-α levels were higher in patients with SD compared with those in patients with DWWS or DWoWS, suggesting that they may be useful prognostic predictors. Among the 45 cytokines tested, CCL5, IL-15, SCF, PDGF-BB, IL-10, and TNF-α levels were associated with disease outcomes. IL-2, TNF-α, VEGF-D, and IL-6 were correlated with thrombocytopenia and these markers were valuable for predicting bleeding. These acute-phase cytokine signatures may therefore be useful for further investigation and identifying patients at risk for the development of SD, particularly on the recent outbreaks in a dengue endemic region, Myanmar.

## Figures and Tables

**Table 1:** Clinicopathological characteristics of the study population.

	DWoWS (*n* = 64)	DWWS (*n* = 64)	SD (*n* = 39)	Total (*n* = 167)
Male	37	45	24	106
Female	27	19	15	61
Primary infection	23	34	7	64
Secondary infection	41	30	32	103
Age: <1 year	0	1	0	1
1–5 years	13	16	2	31
6–12 years	30	37	34	101
13–18 years	11	7	3	21
>19 years	10	3	0	13
DENV RNA-positive	32	33	18	83
DENV-1	10	13	7	30
DENV-3	14	12	7	33
DENV-4	8	8	4	20
DENV NS1 Ag-positive (RDT)	64	64	39	167
IgM positive	50	48	33	131
IgG positive	41	31	33	105

DWoWS = dengue without warning signs, DWWS = dengue with warning signs, SD = severe dengue, DENV-1 = dengue virus-1, DENV-3 = dengue virus-3, and DENV-4 = dengue virus-4, RDT = Rapid diagnostic test.

**Table 2:** Differences in serum cytokine levels between healthy individuals and dengue patients during acute infection.

Cytokines	Control vs. DWoWS	Control vs. DWWS	Control vs. SD	DWoWS vs. DWWS	DWoWS vs. SD	DWWS vs. SD
CCL5	<0.001	<0.001	<0.001	0.044	0.205	0.393
IL-15	0.775	0.211	0.669	0.036	0.788	0.065
SCF	0.003	<0.001	<0.001	0.027	0.311	0.265
PDGF-BB	0.171	0.308	0.017	0.447	0.165	0.028
IL-10	<0.001	0.005	0.002	0.703	0.014	0.261
TNF-⍺	0.079	0.143	0.042	0.642	0.07	0.231
IL1β	0.001	0.002	0.003	0.76	0.277	0.312
IL-5	0.009	0.004	0.007	0.108	0.884	0.051
IP10 (CXCL10)	<0.001	<0.001	<0.001	0.125	0.86	0.188
IL-7	0.006	0.001	0.001	0.055	0.434	0.239
IL-8 (CXCL8)	<0.001	<0.001	<0.001	0.836	0.439	0.223
PIGF1	0.001	<0.001	<0.001	0.133	0.577	0.418
IL-13	0.049	0.049	0.412	0.297	0.608	0.259
IL-17A	0.027	0.018	0.11	0.527	0.535	0.292
MIP-1β	0.002	0.001	0.002	0.461	0.938	0.512
CCL2	0.001	0.001	0.004	0.858	0.401	0.444
VEGFD	0.004	0.004	0.004	0.658	0.556	0.244
NGFβ	0.012	<0.001	<0.001	0.066	0.05	0.883
EGF	0.033	0.014	0.019	0.204	0.841	0.329
IL1⍺	0.001	<0.001	<0.001	0.70	0.718	0.292
IL-23	0.01	0.002	0.002	0.082	0.392	0.310
FGF2	0.007	0.001	0.002	0.071	0.826	0.113

DWoWS = dengue without warning signs, DWWS = dengue with warning signs, SD = severe dengue. Data from more than two groups were compared using MANCOVA, and data between two groups were compared using the Mann–Whitney U test based on distribution of data. *p*–values of < 0.05 were considered as statistically significant. (IL = interleukin, CCL = chemokine ligand, SCF = stem cell factor, PDGF-BB = platelet derived growth factor BB, TNF-⍺ = tumor necrosis factor ⍺, PIGF = placental growth factor, MIP1β = macrophage inflammattory protein 1, VEGFD = vascular endothelial growth factor, NGF β = nerve growth factor, EGF = epidermal growth factor, FGF-2 = fibroblast growth factor).

**Table 3:** Correlations between acute-phase cytokine levels, platelet counts, and NS1 Ag levels in dengue patients.

Cytokine	Spearman’s Rank Correlation	*p*-Value
Correlation with platelet count		
IL-2	−0.019	0.017
IL-6	−0.063	0.039
TNF-⍺	−0.208	0.008
VEGF-D	−0.177	0.025
Correlation with NS1 Ag levels		
IL-1⍺	0.162	0.038
SCF	0.190	0.015
CCL5	0.176	0.024
IFN-⍺	0.336	<0.0001
IL-22	−0.212	0.006

Spearman’s test was used to identify correlations and the results are presented as the coefficients (ρ −1 to +1). *p*–values of < 0.05 were considered as statistically significant.

## Data Availability

The datasets generated during and/or analyzed during the current study are available from the corresponding authors on reasonable request.

## Supplementary Materials

The following are available online at https://www.mdpi.com/article/10.3390/pathogens11050558/s1, Figure S1: Clinical symptoms of enrolled patients, Figure S2: NS1 Ag level and IgM in different groups of severity and viremia level and IgM in different groups of severity. Figure S3. Scree plot to determine the number of principal components (all cases, *n* = 167). Figure S4. The contributions of cytokines to the top 3 principal components denoted by Dim.1, Dim.2 and Dim.3. Figure S5. Clustering of individuals by primary and secondary infection according to principal component Dim.1 and Dim.3. Figure S6. Clustering of individuals by the severity of infection—severe, with and without warning sign (WS) according to principal component. Table S1: Dengue TaqMan RT-PCR serotype primers.
