# Supplementary material for: Acute-phase Serum Cytokine Levels and Correlation with Clinical Outcomes in Children and Adults with Primary and Secondary Dengue Virus Infection in Myanmar between 2017 and 2019
Source: Pathogens. 2022 May 9;11(5):558. doi: 10.3390/pathogens11050558 (PMC9144711; doi:10.3390/pathogens11050558)
Supplement: Supplementary file 1 [file pathogens-11-00558-s001.zip › pathogens-1618454-supplementary-done.pdf]

pathogens

MDPI

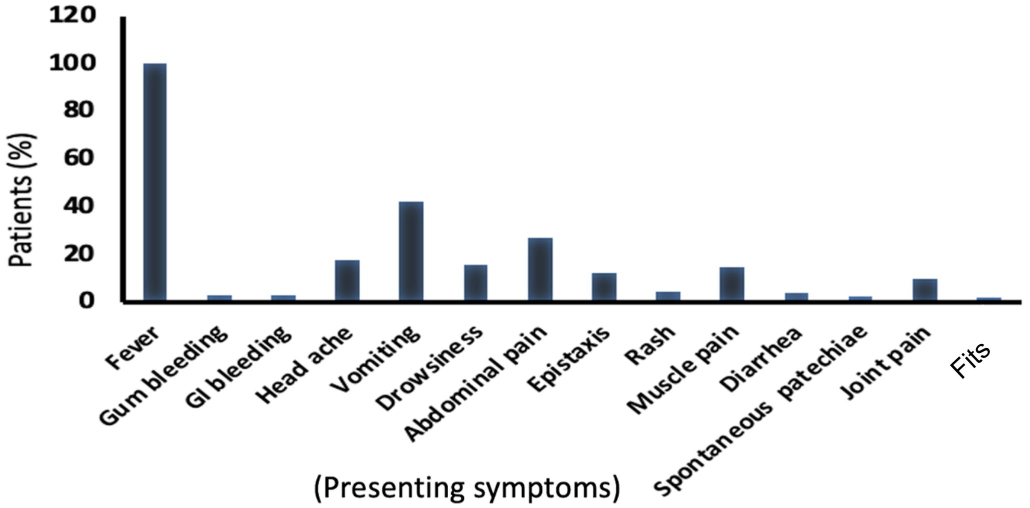

Figure S1. Clinical symptoms of dengue patients in this study.

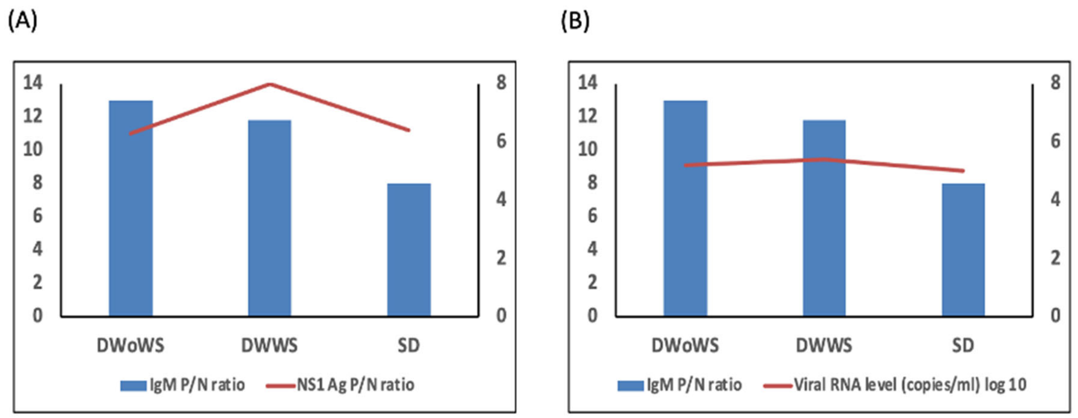

Figure S2. (A) NS1 antigen (red bar) and IgM antibody levels (blue line) in different patient groups according to severity (Dengue without warning signs, DWoWS; Dengue with warning signs, DWWS; severe dengue (SD)).

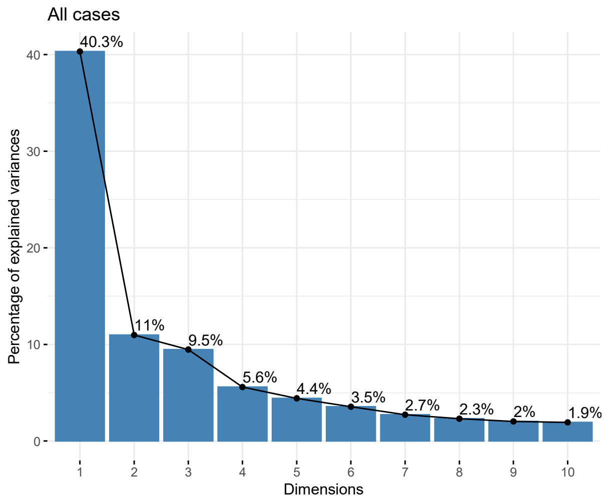

Figure S3. Scree plot to determine the number of principal components (all cases, n=167).

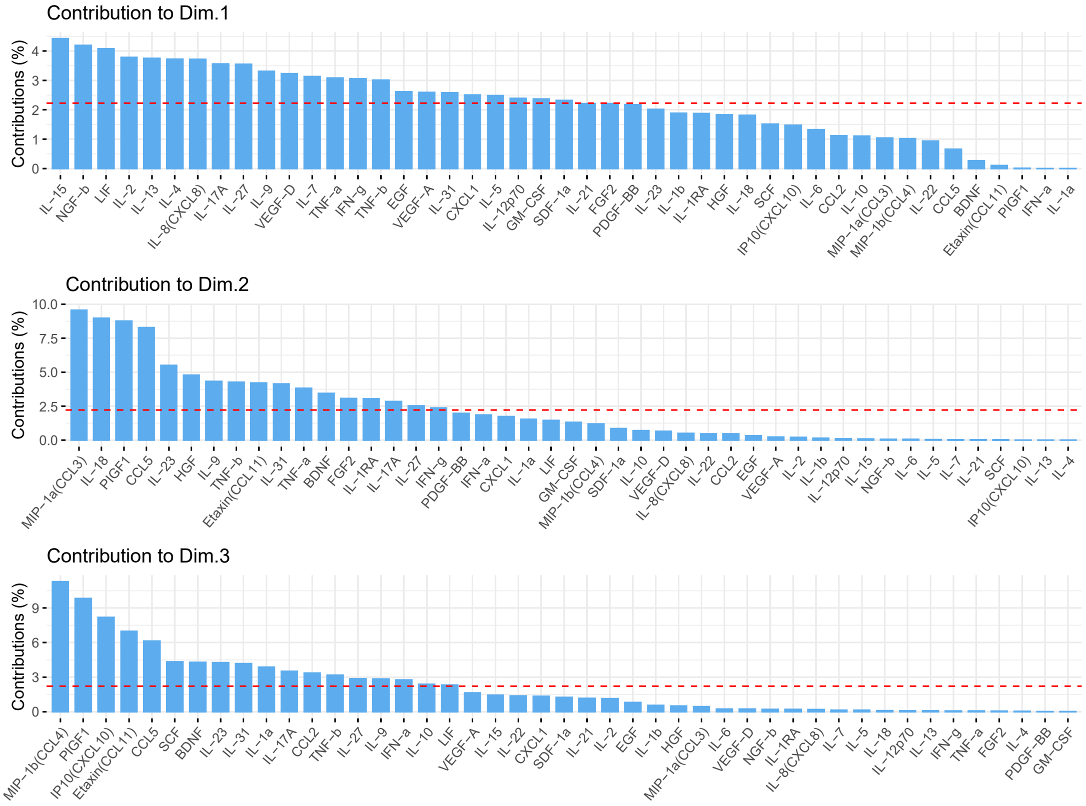

Figure S4. The contributions of cytokines to the top 3 principal components denoted by Dim.1, Dim.2 and Dim.3. All cases (n=167) were used. The horizontal dashed line represents the expected value if the contributions from all of the cytokines were uniform.

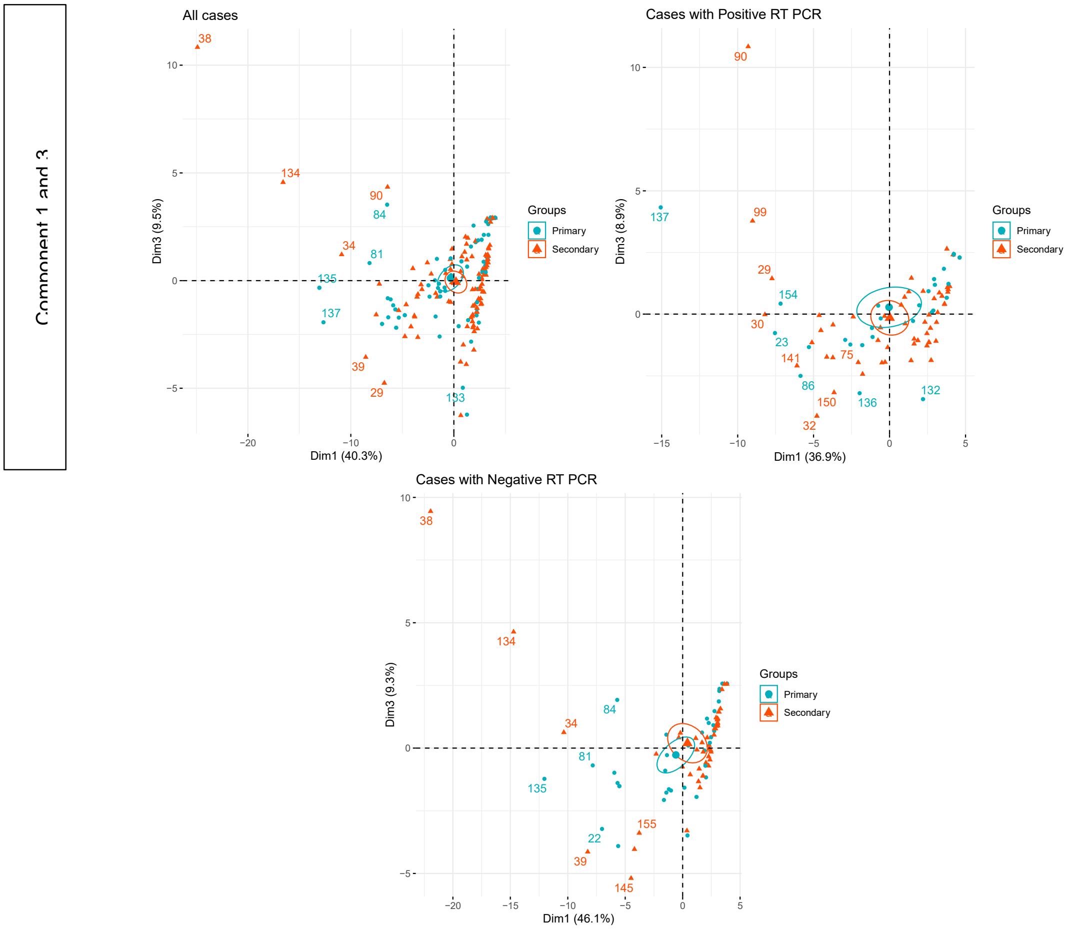

Figure S5. Clustering of individuals by primary and secondary infection according to principal component Dim.1 and Dim.3 for all cases (n=167, first column), cases with positive RT-PCR (n=83, second column), and cases with negative RT-PCR (n=84, third column). Circles represent the group mean.

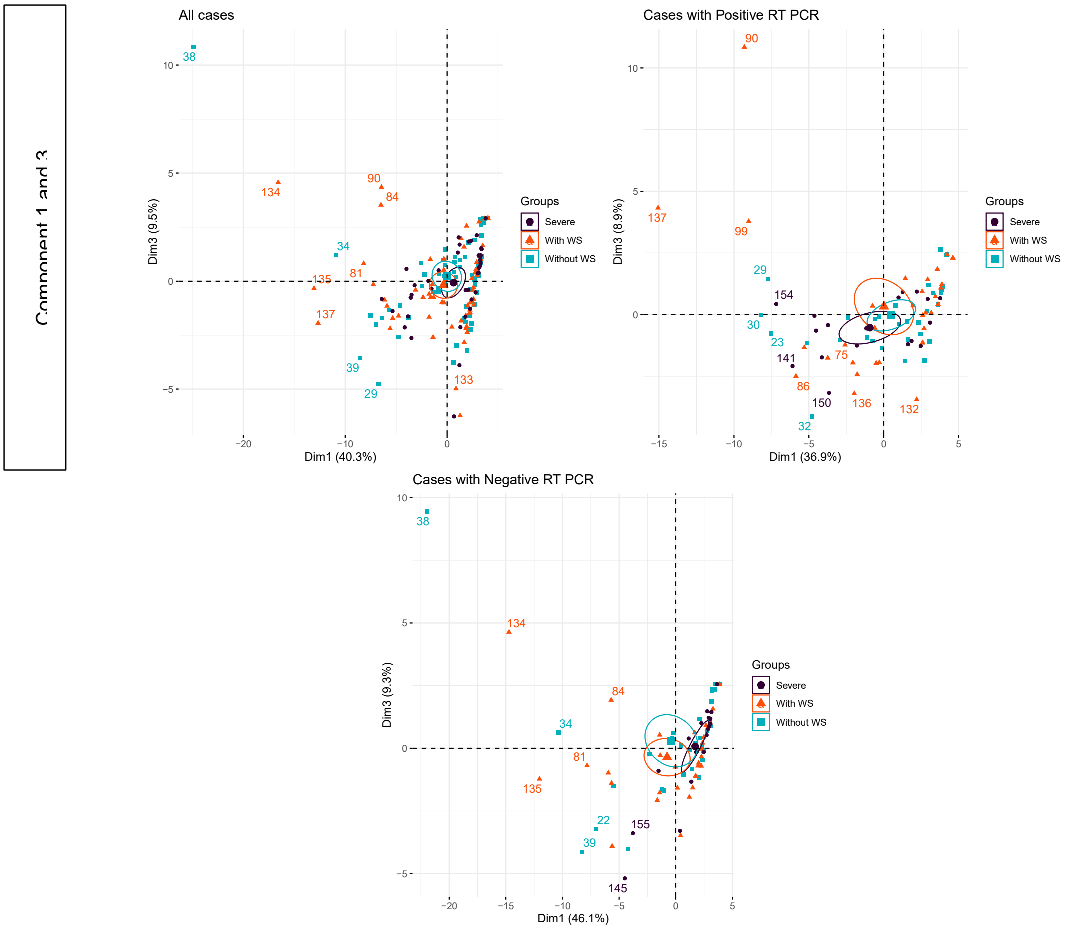

Figure S6. Clustering of individuals by the severity of infection – severe, with and without warning sign (WS) according to principal component Dim.1 and Dim.3 (second row) for all cases (n=167, first column), cases with positive RT-PCR (n=83, second column), and cases with negative RT-PCR (n=84, third column). Circles represent the group mean.

Table S1. Dengue TaqMan RT-PCR serotype primers

Virus	Dengue Serotype Realtime Primer	Sequences (5' - 3')
DENV-1	D1MGBEn469s (forward)	GAACATGGRACAAYTGCAACYAT
	D1MGBEn493p*a* (probe)	ACACCTCAAGCTCC
	D1MGBEn536r (reverse)	CCGTAGTCDGTCAGCTGTATTTCA
DENV-2	D2MGBEn493s (forward)	ACACCACAGAGTTCCATCACAGA
	D2MGBEn545p*a* (probe)	CGATGGARTGCTCTC
	D2MGBEn568r (reverse)	CATCTCATTGAAGTCNAGGCC
DENV-3	D3MGBEn1s (forward)	ATGAGATGYGTGGGAGTRGGAAAC
	D3MGBEn27p*a* (probe)	AGATTTTGTGGAAGGYCT
	D3MGBEn71r (reverse)	CACCACDTCAACCCACGTAGCT
DENV-4	D4TEn711s (forward)	GGTGACRTTYAARGTHCCTCAT
	D4TEn734p*b* (probe)	CCAAGAGACAGGATGTGACAGTGCTRGGATC
	D4TEn786c (reverse)	WGARTGCATRGCTCCYTCCTG
